# Advances and Innovations of 3D Bioprinting Skin

**DOI:** 10.3390/biom13010055

**Published:** 2022-12-27

**Authors:** Moon Sung Kang, Jinju Jang, Hyo Jung Jo, Won-Hyeon Kim, Bongju Kim, Heoung-Jae Chun, Dohyung Lim, Dong-Wook Han

**Affiliations:** 1 Department of Cogno-Mechatronics Engineering, Pusan National University, Busan 46241, Republic of Korea; 2 Department of Mechanical Engineering, Yonsei University, Seoul 03722, Republic of Korea; 3 Dental Life Science Research Institute/Innovation Research & Support Center for Dental Science, Seoul National University Dental Hospital, Seoul 03080, Republic of Korea; 4 Department of Mechanical Engineering, Sejong University, Seoul 05006, Republic of Korea; 5 BIO-IT Fusion Technology Research Institute, Pusan National University, Busan 46241, Republic of Korea

**Keywords:** 3D bioprinting, skin tissue engineering, biomaterials, skin regeneration, wound healing

## Abstract

Three-dimensional (3D) bioprinted skin equivalents are highlighted as the new gold standard for alternative models to animal testing, as well as full-thickness wound healing. In this review, we focus on the advances and innovations of 3D bioprinting skin for skin regeneration, within the last five years. After a brief introduction to skin anatomy, 3D bioprinting methods and the remarkable features of recent studies are classified as advances in materials, structures, and functions. We will discuss several ways to improve the clinical potential of 3D bioprinted skin, with state-of-the-art printing technology and novel biomaterials. After the breakthrough in the bottleneck of the current studies, highly developed skin can be fabricated, comprising stratified epidermis, dermis, and hypodermis with blood vessels, nerves, muscles, and skin appendages. We hope that this review will be priming water for future research and clinical applications, that will guide us to break new ground for the next generation of skin regeneration.

## 1. Introduction

Restoration of damaged skin has been a long-desired goal for modern medicine. Biocomposite dressings have been adopted to improve wound healing in partial thickness wound, and made them the most used strategies for superficial burns and epidermal necrolysis [1]. However, the repair of large-scale deep injuries such as deep burns cannot be handled with conventional wound dressing [2]. To date, full-thickness skin replacement generally adopts graft implantation (e.g., allograft and autograft), but the shortage of skin sources, donor site morbidity, immune reactions, and infection risk limit their use [3]. Moreover, conventional grafts have lowered biological functions and are aesthetically not coordinated with existing tissues, hence, they are unable to recapitulate the full function of skin [4,5]. Therefore, novel approaches based on tissue engineering have been highlighted. The goal of tissue engineering is to develop autologous tissue grafts through a tailored combination of cells and biomaterials, for future full-thickness skin restoration. Various methods to fabricate skin tissue engineering scaffolds have been developed, including foam casting, electrospinning, phase separation, and decellularization [6,7,8,9]. In particular, 3D bioprinting is considered to have a significant impact in the field of tissue engineering, as tissue-scaled large analogs can be fabricated with submicron fidelity [10,11]. Contemporary 3D bioprinted skins have limited use in wound healing applications because long-term stability has not yet been established through many clinical evaluations. However, they have been vigorously used for drug screening platforms within the last decades. To meet the demand for screening platforms without animal testing, in vitro toxicity screening platforms such as skin-on-a-chip have been developed and commercialized. L’OREAL groups (Clichy, France) have developed several kinds of 3D bioprinted skins, including Episkin^TM^ (Clichy, France) and Skinethic^TM^ (Clichy, France), to use for the evaluation of toxicity, irritation, or regeneration of developed cosmetics. Epiderm^TM^ from MatTek (Ashland, MA), Epi-model 24 from J-Tek (Aichi, Japan), and Keraskin^TM^ from Biosolution (Seoul, Republic of Korea) are 3D bioprinted skin tissues which have been widely used for skin irritation testing and drug efficiency screening.

**Figure 1:**
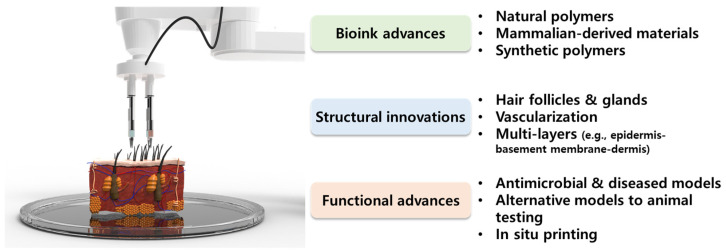
Schematic diagram of recent advances and innovations of bioink materials as well as the structure and function of three-dimensional (3D) bioprinting skin.

The subject matter of this review deals with the advances and innovations of 3D bioprinting skin for skin regeneration, within the last five years (2018–2022) (Figure 1). As most of the reviews do not provide classification of 3D bioprinting skins according to their structure, function, materials, or properties, we intend to provide organized information on which recent novel studies focus on, and what technological advances have been made. The eye-opening progress of recent studies is classified as advances in bioink materials, structures, and functions of 3D bioprinting skin. Natural hydrogels provide a sufficient microenvironment to laden skin cells with excellent printability, while synthetic polymers are often added to strengthen the biological and mechanical properties of bioink. Mammalian-derived materials such as decellularized extracellular matrix (dECM) have been highlighted as they have a high affinity to native tissues, and can mimic the natural niche [12,13,14]. Based on these novel materials, recent studies have focused on the development of structurally advanced skin models such as full-thickness models, hair induction, and vascularized models for drug screening platforms and directly implantable grafts. Thereafter, technological and regulatory hurdles are discussed, which limit the clinical application of 3D bioprinted skin. By providing our perspectives and breakthrough strategies, we hope this review can be priming water for future research and clinical applications, that will guide us to break new ground for the next generation of skin regeneration.

## 2. Skin Anatomy and Functional Replacement of Damaged Skin

**Figure 2:**
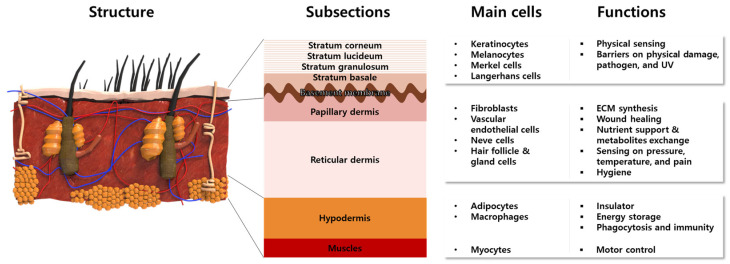
Anatomical structures of multi-layered skin composed of representative cells with intrinsic functions.

The skin is the largest organ of the body, accounting for about 15% of the total body weight in adults [15]. The skin has a hierarchical multi-layered structure that is composed of several components including cells, fibrous matrices, extracellular matrix (ECM), blood vessels, nerves, glands, and hair follicles. Innumerable body functions are regulated by the skin such as physical protection, thermoregulation, sensation, homeostasis, and immunity [16,17]. To perform these tasks, skin mostly consists of three different functional layers, namely the epidermis, dermis, and hypodermis, which are divided into subsections, respectively (Figure 2).

The epidermis is a 50 μm–1.5 mm-thick outermost protective layer, consisting of 90% keratinocytes (KCs) and stratified layers containing different types of cells [18]. The KCs locate at the full layer of the epidermis to synthesize the keratin that constitutes the skin barriers. The melanocytes and Merkel cells at the stratum basale produce melanosomes and sense physical stimuli, respectively [19]. The Langerhans cells are known to serve the immunologic roles of antigen-presenting cells [19]. The lowest epidermis, named the basement membrane, mainly consisted of laminin, to connect the dermis and epidermis. On the other hand, the dermis is composed of 2 layers named the papillary dermis and reticular dermis. The biggest population of cells in the dermis consists of fibroblasts (FBs), which synthesize ECM components such as collagen (Col) and elastic fibers, to provide skin elasticity and mechanical support. Moreover, FBs secrete various cytokines to promote the wound healing process [20,21,22]. The blood vessels exist in the dermis and supply oxygen and nutrients. Along with the arterioles and venules, nerves are widely distributed to sense pressure, temperature, and pain. Hair follicles and sweat glands exist in the reticular dermis to conduct body temperature control and physical buffering, as well as the prevention of microbial activity on the skin surfaces. Beneath the dermis and above the muscle, the hypodermis, which is mainly composed of adipocytes, acts as an insulator and energy storage. The macrophage in the hypodermis is involved in the wound healing process by phagocytosis and adaptive immunity.

The skin often suffers from functional or aesthetic disturbances such as toxic epidermal necrolysis, large chronic ulcers, acute wounds, and burns. Physiological regulation of wound healing is a complex process, relying on many cell types and mediators interacting in a very precise temporal sequence. The repair process includes intercellular interactions, growth factors, and cytokines involved in the restoration process. In general, spontaneous regeneration of the skin requires 28 days, but when the loss is greater than 4 cm in diameter, recovering full-thickness skin is impractical, especially at an older age [23,24]. In addition, deep skin damage and burns cannot be fully recovered, and often leave scars due to changes in the cellular microenvironment, which leads to abnormal cell growth and matrix formation, with a reduction of hair follicles and sweat glands [25,26,27].

## 3. 3D Bioprinting Methods

**Figure 3:**
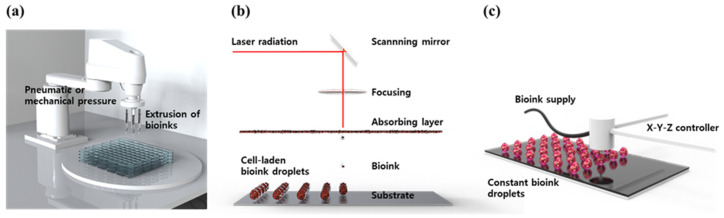
Schematic diagram of common 3D bioprinting methods. (**a**) EBB, (**b**) LAB, and (**c**) DBB.

3D bioprinting is the process of printing biomaterials and cells to produce biological constructs that mimic the characteristics of natural tissues and organs. This is a technology applied by expanding additive manufacturing (AM) technology, to print bio-functional materials in a layer-by-layer (LbL) manner on substrates, after loading cytocompatible biomaterials. 3D bioprinting technology is considered a way to create an ideal 3D structure during in vitro experiments because it can deposit a tailored dose of various cells with spatial manipulation. Using an appropriate 3D bioprinting process, to suit the properties of the material to be used, allows for the proper distribution and positioning of biomaterials, signaling factors, and heterogeneous cells in high densities to form tissue scaffolds. To achieve high cell viability and 3D printability, novel 3D bioprinting technologies such as extrusion-based bioprinting (EBB), laser-assisted bioprinting (LAB), and droplet-based bioprinting (DBB) have been most widely used (Figure 3) [28,29,30].

EBB technology enables finely controlled printing based on automated machine systems and fluid distribution systems [31]. The cell-containing bioink passes through a continuous filament-type micro nozzle in a pneumatic, piston- or screw-driven manner, under computer control. After printing layer by layer, a complete 3D construct is formed. By endowing shear stress, the fine filament structure can be achieved by the EBB method. Recently, EBB has been developed to mount multiple printer heads, which can minimize cross-contamination, while simultaneously depositing various bioink [32,33,34]. Furthermore, they allow better control over shape, porosity, and cell distribution in the printed structure. The advantages of EBB are a wide range of printable bioink types including high-viscosity hydrogel, cell clumps, acellular matrix components, and microcarriers. The most important advantage of EBB is that it can print porous grid structures. The grid structure can promote the circulation of nutrients and metabolites [35,36]. However, there is the disadvantage of low resolution, of which the minimum resolution typically exceeds 100 μm [37,38].

The LAB system consists of four parts: a pulsed laser source, a laser focusing tool, a laser energy-absorbing metal ribbon film, and a receiving substrate [39]. The LAB technology emits laser light through a pulsed laser source, which collects on a metal film on the back of a silicate glass and causes it to be locally heated. At this time, the bioink deposited on the substrate quickly evaporates and is sprayed on the substrate in the form of droplets. As the main energy source, ultraviolet (UV) or near UV wavelengths of nanosecond lasers are used, and the printing resolution is excellent at the level of picogram [40]. In addition, 3D structures printed using these technologies exhibit excellent cell survival rates of 90% or more. Although it has the advantage of being able to print non-contact, showing high cell activity, and printing with high resolution [41,42], it has the disadvantage of not being able to print at high speed, due to the lack of an appropriate fast gelation mechanism.

DBB technology mainly includes Inkjet bioprinting (IJB) and Electrohydrodynamic (EHD) jetting bioprinting. IJB can be divided into continuous inkjet (CIJ) printing and drop-on-demand (DOD) printing. CIJ printing relies on a unique tendency of liquid flow to continuously disperse conductive ink droplets. A CIJ-based bioprinter produces drops at a fast rate. DOD printing generates bioink droplets on the substrate when needed. Thus, it is more suitable for material deposition and patterning with higher precision and minimal waste of bioink [43]. DOD produces droplets, mainly using piezoelectric, thermal, or electrostatic forces, which can flexibly and accurately deposit various biomaterials to construct spatially heterogeneous tissues. However, there are limitations to DOD. First, because the inkjet aperture is extremely small (10–150 μm), it is easily blocked by biomaterials. Therefore, the solvents that can be applied to DOD are only low-viscosity hydrogel or other low-concentration biological agents. Second, since a porous structure cannot be produced, there is a limit to issue perfusion or substance exchange, making it difficult to apply it clinically. EHD jetting bioprinting uses an electric field to pull the bioink droplet out [44]. Therefore, applied voltage, and the physical properties of bioink such as viscosity, and flow rate, have a great influence on cell viability. The EHD method is especially suitable for using highly concentrated bioink [45].

## 4. Recent Advances and Innovations of 3D Bioprinting Skin

### 4.1. Bioink Materials Innovations in 3D Bioprinting Skin

The hydrogel materials for 3D bioprinting were categorized as natural, dECM, and synthetic polymers (Table 1). Natural hydrogels generally provide cytocompatible microenvironments but possess low mechanical properties. Synthetic hydrogels are often used to complement the lowered mechanical properties and endow specific functionalities. dECM bioinks are mammalian-derived materials maintaining plenty of advantageous biomolecules and ECM fibers. The appropriate combination of these materials will promise the realization of highly biocompatible and biofunctional bioinks with suitable 3D printability.

#### 4.1.1. Natural Hydrogel-Based Bioinks

The application of natural polymer-based hydrogel as bioink is advantageous for tissue engineering because oxygen, nutrients, proteins, and other biomolecules readily permeate through the water-swollen hydrogel network to nourish the encapsulated cells. Compared to synthetic hydrogels, most natural hydrogels are much more cytocompatible, and possess rich bioactive amino acid sequences required for cellular adhesion, growth, and maturation. In addition, natural hydrogels are easily decomposed by cellular matrix polymerases, hence, the laden cells can migrate and proliferate. Especially, Alg and Gel are the most widely used bioink materials, due to their excellent cytocompatibility and ease of viscoelastic manipulation. Alg is a family of polysaccharides that can support the growth and proliferation of mammalian cells. The controllable chemical components of Alg (e.g., the content of L-guluronic acid blocks) allow precise control of crosslinking properties and viscosity [56]. Meanwhile, Gel is a denatured Col derived from skin tissues and contains arginine–glycine–aspartic acid (RGD) sequences to support cellular adhesion through integrin interactions [57,58]. By combining the advantages of Alg and Gel, Liu et al. introduced Alg/Gel composite hydrogel bioink to fabricate bilayered membranes for skin tissue engineering [46]. The rheological studies showed that the Alg/Gel hydrogels have temperature-dependent and shear-thinning viscoelastic properties, hence, can be 3D printed in high fidelity. AECs and WJMSCs were encapsulated in the Alg/Gel bioink and could be 3D printed in geometrically complicated forms such as a tube, cylinder, box, nose, and ear-like constructs while maintaining high cell viability (up to 95%). Furthermore, microarray analysis of global gene expression of AECs and WJMSCs revealed that the AECs and WJMSCs showed increased expression of genes involved in cell junction assembly, cell adhesion, epidermis development, wound healing, and the vascular endothelial growth factor (VEGF) signaling pathway. AECs expressed high doses of keratins, indicating their epithelial cell phenotypes. The genes of WHMSCs were upregulated especially with skin regeneration and angiogenesis, suggesting its superior angiogenic potential and fibroblastic phenotype.

**Figure 4:**
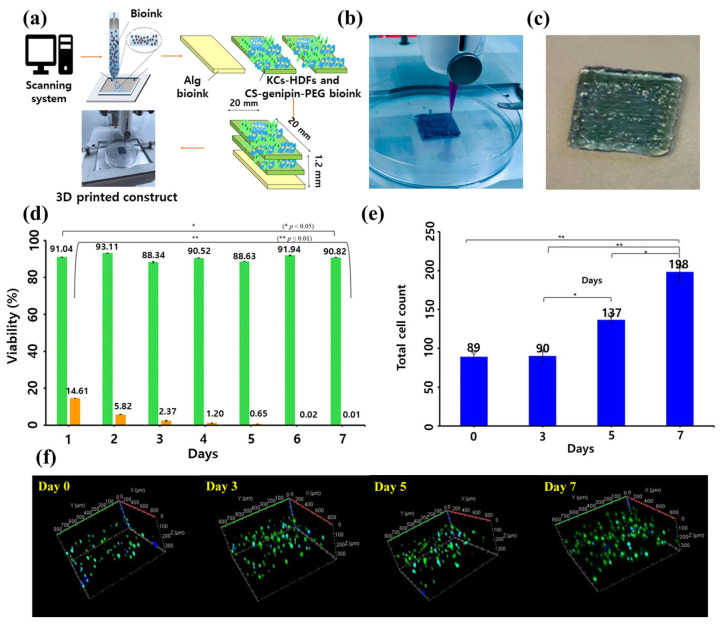
CS-genipin-PEG bioink laden with human KCs and HDFs for skin bioprinting. (**a**) Schematic diagram of the 3D printing process and the fabricated skin constructs. Digital image on (**b**) 3D printing process and (**c**) printed skin constructs. (**d**) MTT assay on the bioprinted skin constructs. The CS-genipin-PEG is green, and TritonX-100 (positive control) is orange. The cell viability was assessed by % of negative control (untreated). (**e**) Total cell count on the printed constructs during 7 days of culture and (**f**) 3D CLSM images. The asterisks (* and **) refer to significant differences (* = *p* < 0.05; ** = *p* ≤ 0.01). The data were reproduced from Ref [47]. Copyright 2020 MDPI.

On the other hand, CS is a semi-crystalline polysaccharide copolymer of glucosamine and N-acetylglucosamine connected by a β (1–4) linkage [59]. It is mainly obtained from the partial deacetylation of chitin, under alkaline conditions or by enzymatic hydrolysis [60]. For skin tissue engineering, CS is an excellent wound healing material due to its excellent biocompatibility, biodegradability, wound healing ability, hemostatic properties, and antimicrobial properties [61,62]. Hafezi et al. introduced CS-genipin bioink laden with human KCs and HDFs for skin tissue engineering applications [47] (Figure 4). A reproducible and reliable 3D printing process was established by applying low pressures (20–40 kPa) that induced 93% cell viability up to 7 days of culture. Owing to the macropores within the constructs and sufficient transfer of nutrients, the high viability of the embedded cells was maintained.

Pectin, a heteropolysaccharide extracted from the cell walls of plants, has excellent biocompatibility and ease of functional group modification [63]. Moreover, physical crosslinking is available through the interaction of calcium ions and blocks of galacturonic acid residues [63]. Basically, the key properties of pectin are comparable to Alg, but its branched nature and complex chemical composition provide versatile chemical modification to have characteristics such as bioadhesive, anti-cancer effects, and immunomodulatory effects [64,65,66]. Pereira et al. introduced RGD-functionalized PECMA bioink, which is dual-crosslinkable with UV photopolymerization and ionic gelation [48]. The laden HNDFs exhibited high cell viability and typical spindle-shaped morphology after 7 days of culture. As UV radiation time and polymer concentration change, the stiffness was PECMA hydrogel was sharply tailored and induced matured morphology and spreading of HNDFs, after 14 days of culture. At this time, fibronectin, Col I, and laminin were sufficiently produced within the constructs, suggesting the cultured dermal construct resembles the ECM composition of native dermal tissues.

#### 4.1.2. dECM-Based Bioinks

As the skin ECM is composed of functional molecules secreted by the resident cells and native filament structures, the dECM hydrogel can retain various molecular components found in native tissues, such as cell adhesion proteins, growth factors, and glycosaminoglycan (GAG). These substances support a constructive and site-specific remodeling response, when used as skin tissue engineering materials. Moreover, unlike xenografts and allografts, dECM materials do not induce host inflammatory and immune responses, due to the absence of cellular antigens [67]. The focus of the dECM hydrogel is the complete removal of residual cells, while maintaining the native biomolecules and ECM components. To fabricate the dECM hydrogel, chemical, enzymatic, and physical approaches have been introduced to efficiently remove the residual cells within the extracted tissues [68,69].

Bera et al. used the goat dermis-extracted dECM bioink for 3D bioprinting of skin mimetics [49]. The single-stem Hypotonic/Hypertonic (H/H) NaCl solution was used to decellularize the goat skin, which resulted in a high yield of residual DNA content, and GAG and Col contents. The constructs of 2 × 2 cm size with micron-sized lattices were 3D printed with high fidelity, due to the excellent shear thinning and shear recovery properties of the synthesized dECM bioink. High cell viability, normal proliferating rate, and spindle-like morphology of L929 cells were maintained during 7 days of culture. The applied shear stress during the printing process induced the alignment of ECM proteins that induced 78% of cells to be isotropic. It is suggested that the isotropic arrangement can enhance the structural and functional integrity of the laden cells which is observed in native ECM fibers [70]. On the other hand, Jorgensen et al. introduced fibrinogen-incorporated dECM bioink for full-thickness wound regeneration [50]. Fibrinogen is a biocompatible and biodegradable natural polymer involved in blood coagulation and wound healing process. In long-term observations in vivo, the implanted dECM-based skin constructs tend to build an immature ECM, whereas the native human dermis has rich Col and fibril structures. This problem can be compromised by the addition of fibrinogen. By the extrusion method, the HDFs-laden fibrinogen/dECM bioink was 3D printed in various geometry, at millimeter scales. The dECM components strengthen the mechanical and shear thinning properties. At 1 and 8 days of culture, the HDFs in both fibrinogen and fibrinogen/dECM bioink showed high viability (85–90%), however, that of fibrinogen decreased to 55% because of the lowered mechanical properties of the hydrogel. Therefore, it is suggested that the long-term viability of laden cells can be retained by the combination of dECM and fibrinogen.

The regenerative cells of adipose tissue include stem cells, angiogenic progenitor cells, pericytes, vascular smooth muscle cells, and immune cells. These cells are called a stromal vascular fraction (SVF), which provides paracrine effects on angiogenic, immunomodulatory, and inflammatory modulatory effects [71,72]. The micronized adipose tissue (i.e., microfat), obtained from the mechanical treatment of lipoaspirate, maintains SVF and can enhance angiogenesis and cytocompatibility [73,74]. Schmitt et al. reported a closed-loop fat processing system, called MiniTCTM, that can process the lipoaspirates into microfat clusters involving the viable cells of SVF [51]. The extracted microfats were mixed with ColMA. The 2 cm lattice disks with 1.5 mm line fidelity were 3D bioprinted and maintained their structure within 7 days of culture regardless of microfat contents. The cryopreserved MSCs, EPCs, and ASCs in the microfat showed high viability. The Alamar blue assay indicates that the cell metabolic activities were significantly increased as the fat concentration increased. The wound healing cytokines including interleukin-6 (IL-6), interleukin-8 (IL-8), FBs growth factor-2 (FGF-2), macrophage colony-stimulating factor (M-CSF), and MIG/CXCL9 are regulated upon activation. Normal T cell expressed and, presumably secreted RANTES, were significantly up-regulated in the microfat-included ColMA hydrogel. These results suggest that the microfat-laden Col constructs release pro-inflammatory cytokines during the in vitro culture.

Meanwhile, the DSIS is a naturally biodegradable dECM that was first prepared by mechanical decellularization of porcine small intestinal submucosa extracts [75]. It can serve as a potential candidate for wound healing and repair and skin tissue engineering, owing to its high biocompatibility, wound healing ability, and tissue regeneration potential [76,77]. Shi et al. prepared the DSIS slurry for potential applications in skin tissue engineering. The DSIS bioink was prepared as a series of decellularization, lyophilization, cryomilling, and dissolution [52]. The prepared DSIS slurry maintained 54% of GAG and 77% of Col, compared to extracted porcine small intestinal submucosa. Through cryogenic 3D printing, FBs-laden DSIS slurry was 3D printed into 80-layered 2 cm-high lattice constructs with micron-sized lattices. The live/dead staining indicates that the laden FBs high cell viability which normally proliferate during 7 days of culture. The western blotting and rtPCR results revealed enhanced expression of VEGF, Col I, Col III, and fibronectin suggesting that the DSIS can accelerate epithelization and wound healing.

#### 4.1.3. Synthetic Hydrogel-Based Bioinks

Synthetic polymer hydrogels can modify the physicochemical characteristics by chemical structures, synthesis methods, water contents, and crosslinking. Any changes in chemical composition can lead to possessing optimized properties as bioink material. In recent studies, the excellent mechanical properties of synthetic polymers can reinforce the elasticity or stiffness of natural hydrogels. Zou et al. developed agarose/nanocellulose/Alg hydrogel bioink with a PVA sacrificial layer to fabricate the large-scale constructs [53]. The HUVECs and HFBs were laden in the bioink and tissue-scale human face-like skin constructs were fabricated. The live/dead assay showed that the laden cells maintained high viability, up to 42 days of culture. DNA contents, Col synthesis, IL-6, and transforming growth factor- β1 (TGF-β1) were increased during 14 days of culture, suggesting that wound healing and neovascularization were facilitated. IF staining of CD31 and vimentin indicates that the HUVECs and HFBs grow around the fluid channels, forming continuous vascular-like structures, showing a vasculatoid prototype. The results suggest great potential for a PVA supporting layer for the engineering of large-scale, complex, and vascularized tissue for face printing.

A typical study uses an EBB method to minimize stress applied to cells, but other methods are also used to fabricate more precise structures. Especially, it is still very difficult to recapitulate the hierarchical porous structure found in most natural tissues. Ng et al. used a single-step DOD bioprinting strategy to fabricate hierarchical porous hydrogels [54]. By increasing the PVP concentration in the Col-based bioink (which is referred to as fractional volume occupancy, FVO), the fiber diameters and micropores of the hydrogel also increased. In the high FVO, fibronectin deposition significantly increased, but the proliferation rate slightly decreased, due to the large pore sizes. Thereafter, a structure having a six-layer hierarchical structure was manufactured by adjusting the number of droplets and FVO. As a result, hydrogels with continuously different pore sizes in the top, middle, and bottom layers were fabricated, which mimics the native skin ECM, supposed to be used in the fabrication of future full-thickness skin models.

On the other hand, the combination of electrospun nanofibrous matrices and 3D printed hydrogels could be an excellent strategy to mimic the epidermis and dermis layers. Miguel et al. fabricated a skin asymmetric construct using electrospinning and 3D printing [55]. The top PCL/SS electrospun membrane mimics epidermis properties, while the bottom layer composed of CS/Alg hydrogel reproduces the dermis layer. Both on PCL/SS matrices and in CS/Alg hydrogel, the HDFs showed high viability and proliferation, during 7 days of culture. Moreover, common spindle-like morphology of HDFs was observed by SEM, and the cells migrated into the hydrogel interior through the hydrogel pores. Especially, the top PCL/SS layer provides microbial protection against *S. aureus* and *P. aeruginosa*, owing to the antibacterial properties of SS. Moreover, the charge effects of CS in the bottom CS/Alg hydrogel also showed excellent antibacterial efficiency (90.91% to *S. aureus* and 66.85% to *P. aeruginosa*), suggesting their future use in wound dressing in the prevention of microbial infection.

### 4.2. Structural Advances in 3D Bioprinting Skin

In this section, we present the current advances and innovations of 3D bioprinted skins, especially on their structural advances including hair follicle, and skin vascularization, and discuss future development direction and clinical applications (Table 2). Development of skin appendages, vessels, and full thickness models will provide more realistic skins to mimic the native functions and physiology of human skins.

#### 4.2.1. Hair-Developed Skin Equivalents

The skin is a complex organ consisting of rich appendages including the hair follicle, nail, and sebaceous gland. Especially, the hairs support a barrier against the penetration of harmful UV rays from the sun and invasion of harmful pathogens, as well as maintain the body temperature and immaculate skin surface. As the contemporary grafts and engineered skins lack appendages, the regenerated scars are conspicuously different from the original intact skin, reducing an individual’s quality of life due to their appearance. More importantly, none of the commercialized skin models could implement hair structures, and drug screening for the treatment of alopecia, folliculitis, and scalp diseases are limited. To address the issue, Kang et al. developed the hair follicle-developed 3D bioprinted skin construct using GelMA/HAMA hydrogel bioink [23]. The printed constructs have an epidermis-papillary layer-dermis triple-layered structure, the papillary layer, which supports epidermis-dermis interaction by increasing surface area. As the ratio of GelMA and HAMA reappeared the ratio of Col and GAG in the natural skin tissues, the laden HDFs and HaCaT cells could spread and show high cell viability. Moreover, the laden HFDPC spheroids maintained proliferating and hair-inducing properties, which spontaneously developed hair pore structures. Although further studies on in-vivo efficiency evaluation are needed, the 3D printed skin equivalent could be a novel approach for hairy skin engineering. Yang et al. introduced the recombinant human Col bioink to fabricate the 3D printed epidermis-dermis double-layered skin mimetics [78]. GelMA and rhCol3 were laden with HaCaT cells and HDFs. The dermal constructs promoted high viability and spreading of laden HDFs, as well as increased expression of Col I, TGF-β, vimentin, and α-smooth muscle actin (α-SMA), suggesting the FB proliferation and keratinocyte differentiation in skin tissue [89,90]. At every ratio of GelMA and rhCol3, high printing fidelity was observed, and the live/dead assay shows that almost 100% of cells are viable and proliferative. Gene expressions of P63, filaggrin, and Nrf2 were significantly upregulated in GelMA-rhCol3, compared to pristine GelMA, which are markers of HaCaT cell differentiation and re-epithelialization [91,92]. After 14 days of rat transplantation, fresh hair follicles were also found in the regenerated dermis of GelMA-rhCol3 bioinks, implying the role of Col for in vivo regeneration.

**Figure 5:**
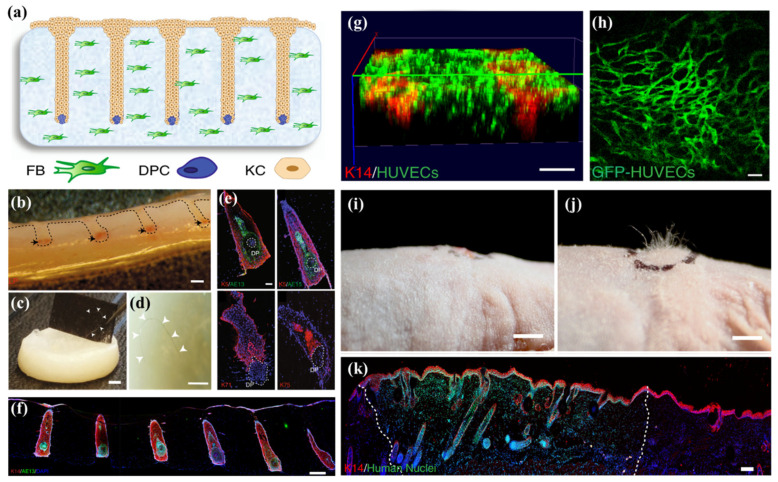
Hair follicle-developed skin constructs by the over-expression of Lef-1. (**a**) Schematic diagram on the fabrication of skin constructs using 3D printed micro papilla mold. (**b**) Cross section of the skin constructs exhibiting ALP (black arrows). (**c**,**d**) Prolonged in vitro culture led to hair fiber formation and protrusion. (**e**) IF staining on the HFU shows the expression of HFU-specific markers. (**f**) Expression of AE13 in HFU, comprised with Lef-1-transfected HFDPCs, showing highly efficient hair differentiation. (**g**) Capillary-like networks after 3 days of culture. (**h**) Explanted pre-vascularized skins, indicating host vascularization and blood supply to the grafts. **(j**) The human hair growth on the nude mouse skin after 4–6 weeks of transplantation, and (**i**) control. (**k**) The human nuclear staining on the explanted grafts. The boundaries (dashed line) between mouse and human tissues are revealed. The sizes of scale bars are as follows: (**b**–**d**,**g**,**i**,**j**) 2 mm, (**e**,**h**) 100 µm, (**f**) 300 µm, and (**k**) 200 µm. The data were reproduced from Ref [79]. Copyrights 2018 Nature Publishing Group.

On the other hand, Abaci et al. introduced a hair follicle-developed skin construct that enhances the efficiency of hair follicle differentiation [79] (Figure 5). A 3D printed micro papilla mold was prepared to create the microwells in the HFBs-laden skin constructs. Subsequently, the HFDPCs were seeded in the microwells, and KCs were added on top of the gel. Interestingly, an HFU-like unit and hair shaft were developed at in vitro culture exhibiting alkaline phosphatase (ALP), versican, K5, K71, K75, and AE13, which are markers of anagen phase hair follicle, inner root sheath (IRS), outer root sheath (ORS), and companion layer, implying the development of functionally and structurally matured hair follicles [93,94]. The development of the hair shaft is attributed to the overexpression of Lef-1 in HFDPCs that restore intact transcriptional signatures and hair follicle differentiation. The in vivo transplantation of engineered skins induced human hair growth in immunodeficient nude mice, implying their impact on the medical management of alopecia.

#### 4.2.2. Vascularized Skin Equivalents

Another challenge to achieving the successful clinical translation of bioengineered skins is vascularization. Most current skin grafts composed of FBs and KCs lack dermal vascular networks important for perfusion, complicating long-term graft survival. Baltazar et al. introduced 3D bioprinted implantable xeno-free vascularized human skin grafts. Plenty of cells including HECs, PCs, and KCs were laden in the xeno-free dermal and epidermal bioink, reinforced with PGA mesh to increase structural stability. After 4 weeks of transplantation to nude mice, the presence of human PCs in the dermal layer promoted a mature stratified epidermis with rete ridge-like structures. Moreover, the in vivo experiments showed the developed perfused microvessels preventing graft necrosis and eliciting further perfusion of the graft by angiogenic host microvessels. Through the xeno-free approach to complex tissue engineering, the incidence and severity of allograft rejection could be significantly reduced in clinical skin transplantation. Turner et al. reported a core-shell extrusion 3D bioprinting strategy to fabricate the vascularized skin constructs [81]. Despite the excellent wound healing and angiogenic properties of DA and CS, the poor mechanical strength limits their use in skin bioprinting. Therefore, the study used GelMA to enhance the 3D printability and mechanical properties. The HUVECs-laden DA/CS core and BMSCs-laden shell lattices were pneumatically 3D printed. Dual peptide P15/MMP-2 was incorporated in the DA/CS core to enhance cellular behaviors. The IF staining after 12 days in vitro resulted in cord-like natural micro-vascularization as tube-like structures with endothelial cell marker expression being confirmed. Furthermore, the ARS staining indicated that the bioink induced osteogenic differentiation of the cells, suggesting enhanced in vitro skin wound healing activity and maintained multipotency of BMSCs. On the other hand, challenges including a narrow range of printable material viscosity (typically from 3.5 to 12 mPa/s) and printable viscosities (typically from 1 to 300 mPa/s) are the main hurdles in extrusion-based 3D bioprinting technologies. Especially, the micro-structured vessels are hardly recapitulated by most extrusion-based 3D bioprinting, due to the limited printability. However, DLP-based 3D printing technology can be a breakthrough in printing complex structures with higher printing speeds, microscale resolution, and cell viability [95,96,97]. Zou et al. reported GelMA/HA-NB/LAP bioink and DLP 3D printing of vascularized dermal constructs [82]. The submicron-sized lattices were 3D printed and supported high viability and proliferation of HFBs and HUVECs, during 5 days of culture. The skin constructs formed interconnected microchannels and facilitated cell migration and adhesion outside the bioprinted skin. Furthermore, inflammatory cell infiltration wound healing and angiogenic markers expression were significantly enhanced in rat and pig models. The instant defense function and dermal regeneration were achieved with the developed skin appendages, suggesting the neovascularization of the skin analogs in supporting the development of the physiologically akin structures with native skin. Baltazar et al. introduced the fabrication of implantable multilayered vascularized 3D bioprinted skin grafts [83]. The bioink is composed of Col I from rat tails including HFBs, HUVECs, HECFCs, PCs, and HKCs. On in vitro culture, matured KCs formed a multilayered barrier, while the HUVECs and PCs were self-assembled into interconnected microvascular networks. The IF staining and IHC analysis showed that re-epithelialization and ECM production were achieved in the constructs. The grafts were implanted on to the mice formed perfused microvessels and epidermal rete after 4 weeks. These results imply that the HECFC-derived HUVECs and placental PCs can be used to promote the vascularization and perfusion of bioprinted skin grafts.

#### 4.2.3. Full-Thickness Skin Equivalents

Unlike conventional skin grafts, recently developed full-thickness skins recapitulate multi-layer structures and appendages using 3D bioprinting. Molnar et al. fabricated epidermis-dermis-hypodermis skin mimetics using the Gel/glycerol/HA composite hydrogel bioink [84]. HKCs, dark-melanocytes, HDFs, HDMECs, HFDPCs, and preadipocytes were suspended in fibrinogen bioink and bioprinted to form a 2.5 × 2.5 cm triple-layered patch with micron-sized lattices. After observation of total wound closure with re-epithelialization in vivo, the skin samples were harvested at 21 days for IHC analysis. The bioprinted skin showed less aligned Col structures compared to the control hydrogel-treated wound, suggesting more normal and basket weave Col of native skins was developed. Moreover, the transplanted human cells were normally grown in the regenerative dermis while stratified epidermis, dermal maturation, and blood vessel formation were observed in the bioprinted skin. Therefore, the results imply that the remodeled skin resembles human skin. Barros et al. utilized HDFs, HUVECs, and HKCs-laden GelMA/Alg bioink to fabricate the epidermis-dermis with vascularized skin structures [85]. The 3D printability and cell viability were optimized at differing blending ratios of GelMA and Alg. It was suggested that the controlled matrix stiffness regulated pro-Col1α1 and MMP-1 expression in HDFs, and affected their viability, proliferation, and spreading. Moreover, the multiple seeding of HKCs, on the top of the dermal constructs, at 135 µm-sized stratified epidermis, was fabricated with high cell viability. Somasekharan et al. used Alg/Gel/DCEL bioink to fabricate epidermis-dermis double-layered skin mimetics [86]. Using extrusion-based 3D bioprinting, the micron-sized highly fine lattice-structured cylinder model was fabricated. The Alg/Gel/DCEL bioink exhibited excellent printability, non-cytotoxicity, and stable hydrogel formulation. The incorporated DCEL induces uniform distribution of cellulose fibers within bioinks, supporting excellent cell viability of the laden HFBs and HKCs. After 21 days of culture, CK14 in the dual-cell laden constructs and Col in the dermis were observed showing Col synthesis and skin-specific markers were expressed in the biomimetic skins. Min et al. reported a full-thickness skin model containing pigmentation of MCs that is the first developed engineered ephelides in biomimetic skin [87]. After repeated accumulation of HDFs-containing dermal layers, MCs and HKCs were sequentially 3D printed on top of them to induce skin pigmentation, upon subsequent air-liquid interface culture. The bioprinted skin structures showed matured dermal and epidermal layers, as well as terminal differentiation of KC, forming the stratum corneum. Moreover, the MCs in the epidermal layer showed a spontaneous differentiation to the freckle-like pigment within the skin-epidermal junction, without any external stimuli. The melanogenesis and formation of visible pigments can be contributed to the use of high MC densities and additional time for MCs to grow in the dermal layer. In the other study conducted by Derr et al., a full-thickness skin equivalent model was fabricated, and their barrier functions were evaluated [88]. The dermis layer was fabricated using Gel/Col/elastin composite hydrogel with HDFs. The basal layer was fabricated using laminin/entactin with a jetting dispenser to facilitate the application of a thin uniform layer above the dermal layer, and subsequently, HKCs were seeded on top of the constructs. The H&E staining of the matured skin equivalents showed a fully differentiated epidermis showing stratum corneum, stratum granulosum, stratum spinosum, and stratum basale structures. The IHC staining showed developed tight junction, ECM proteins, and proliferation markers. Moreover, the stratum granulosum formed an f-TKD shape, allowing homeostasis by a tight junction barrier. The impedance measurements, invasion assays, and cell viability assays show viable tissues with good barrier function and good reproducibility. The fully matured epidermis structures are suggested to be commercially applied in the robust testing of compounds, for toxicity effects and the development of skin disease models for drug testing.

### 4.3. Functional Advances in 3D Bioprinting Skin

In this section, recent functional developments of skin equivalents and 3D printing technologies were reviewed (Table 3). For prevention of anti-microbial infection of tissue transplantation, anti-microbial skins were developed. Diseased models were introduced to mimic the patology and microenvironments of diseased skins, while the in situ bioprinting platform enabled more realistic approches in clinical bioprinting.

#### 4.3.1. 3D Bioprinting Skin Equivalents as Antimicrobial and Diseased Models

Despite significant advances in tissue-engineered skins, the risk of bacterial infection at the lesion site remains the main challenge [98,108]. The development of bioinks with strong antibacterial activity, which serve as a physical barrier for cells, is highly valuable for preventing infections associated with impaired wound healing [109,110]. However, the highly hydrated environment of the hydrogel makes them vulnerable to microbial infection. To address the issue, Rastin et al. reported the 3D bioprinting of skin equivalents with antibacterial polysaccharide composites [98]. The Alg-based bioink is reinforced and crosslinked with MeC and Ga. Ga is demonstrated to have antibacterial activity toward many Gram-negative and Gram-positive bacteria, through the disruption of iron-dependent metabolic pathways by bacterial cells, due to the chemical similarity of Ga^3+^ and Fe^3+^ [111,112,113]. The bioink included HFBs and 3D printed into square (h = 4 mm) and round (h = 13 mm) disks with micron-sizes inner lattices by an extrusion-based method. The highly thixotropic behavior of the bioink allows the fabrication of multi-layered structures with excellent 3D printability. The presence of Ga cations on the hydrogel surface exhibited antibacterial activity toward *S. aureus* and *P. aeruginosa*, while the laden HFBs maintain high cell viability during 48 h of culture. Therefore, the use of Ga-crosslinking in skin tissue bioink could be a great strategy for antibacterial activity and high cytocompatibility. Meanwhile, several antimicrobial drugs have been used in 3D bioprinting skins to accelerate the wound healing process. Within them, Nafcillin is a penicillinase-resistant penicillin that inhibits the crosslinking of cell wall peptidoglycan, especially useful for the treatment of infection caused by methicillin-sensitive penicillinase-producing staphylococci [114,115]. Si et al. introduced Nafcillin-loaded HA-MA/HA-SH dual crosslinkable bioink for skin tissue engineering applications [99]. The HA-MA hydrogels have high swelling rates and fast decomposition, while HA-SH has excellent biocompatibility [116]. The HDFs were incorporated into the bioink and 3D printed to a 1 × 1 cm construct with millimeter-sized lattices. The live/dead assay and CCK-8 assay showed that the laden HDFs exhibited high viability and proliferation within 7 days of culture. The in vitro drug release assay showed that the loaded Naficillin has a prolonged release time, one that is consistent with the swelling behaviors of the hydrogel.

Until now, few disease models demonstrating the pathological processes present in native skin have been reported. Kim et al. reported the 3D bioprinted disease skin analogs with pathophysiological features of type 2 diabetes in vitro [100]. The dECM bioink composed of the porcine dermis, hypodermis, and vascular tissues was used with the PCL transwell system to fabricate the full-thickness skin equivalents composed of the epidermis-dermis-vascular channel, and hypodermis. NHDFs and NHPAs were used to fabricate the normal dermal compartments, while dHDFs and dHPAs were used to fabricate the diabetic dermal compartments. IF and IHC staining showed that the normal tissues showed high cell viability with epithelialization, ECM production, and vasculature. On the contrary, slow re-epithelization, insulin resistance, adipocyte hypertrophy, inflammatory reactions, and vascular dysfunction were found in the diabetic skin model, which are typical hallmarks of native diabetic skin. The normal HKCs interacted with diabetic dermal compartments and could differentiate into diabetic epidermis, by stimulating epidermal-dermal intercellular crosstalk found in the native skin. It is suggested that the development of morphologically and physiologically accurate in vitro diseased models could offer a new solution to resolve diabetes-related skin complications.

#### 4.3.2. In Situ 3D Bioprinted Skin

A new strategy of 3D bioprinting, in situ bioprinting, was first proposed in 2007, which refers to the creation or recovery of living skin tissue by directly printing bioink on the defective areas [117]. The hand-held bioprinting system is one of the most used in situ printing strategies. The hand-held bioprinter does not require defect scanning or computer-aided path selection, and lets the operator simply grasp the bioprinter to deposit the bioink directly onto the site by moving the hand. This strategy provides significant freedom to the clinician to adjust dressing shape according to the operation conditions, such as patient movement or complicated wound shape, that cannot be conveniently handled by the robotic arm method. Ying et al. reported on an open-source hand-held extruder loaded with pore-forming bioink for in situ wound dressing applications [102]. Because the inner pore sizes of the hydrogel can modulate cell behaviors such as their spreading, proliferation, and migration, it was sharply tailored by control of the mixing time and ratio of GelMA and PEO. Compared to the control, the pore-forming GelMA/PEO hydrogel induced increased cell spreading and proliferation of NIH/3T3 and HUVEC cells. It was suggested that the pore-forming bioink enhanced liquid and oxygen transport that supported the growth of laden cells. Moreover, different-shaped scars were fully dressed up showing high reproducibility of hydrogel structures up to 50 cycles and high cell viability up to 10 cycles. In the other study conducted by Cheng et al., a handheld 3D bioprinter, used to fabricate the one-step in situ formation of cell-containing skins, that accommodate the topography of the wound, was used [103]. The fibrin was incorporated into the HA hydrogel to facilitate the epithelialization process and recruit host cells during the wound healing cascade [118,119]. The phenotypes of the MSCs were maintained, which allowed their differentiation into adipogenic and osteogenic lineages. In addition, the MSCs-containing fibrin/HA bioink was directly injected on the porcine full-thickness burn injury. After 28 days, in the MSC and fibrin/HA transplanted group, epithelization speed and thickness were significantly increased, while the scar scale and contracture were decreased. There was a significant increase in epithelialization speed, a reduction in scarring, as well as a reduction in contracture formation, per wound area. In addition, typical markers of vascularization were up-regulated, while inflammatory cell surface markers were lowered, suggesting the attenuated immune response. These studies suggest that the developed hand-held 3D printing system could be used in rapid and effective in-surgery wound healing applications.

Compared to hand-held extrusion methods, robotic arm methods provide higher anatomical precision to achieve precise spatial deposition of the dressing biomaterials. Zhao et al. developed PRP-integrated Alg/Gel hydrogel bioinks to print bilayered skin tissues and evaluate the biological effects in vitro and in vivo [104]. The PRP is the plasma containing high concentrations of platelets that accelerate the hemostatic process by promoting thrombosis and coagulation in wounds [120,121]. In in vitro assays, the PRP/Alg/Gel bioink supported high cell viability, proliferation rate, and migration of HDFs and HESCs. The representative ECM components including Col I, Col III, decorin, and fibronectin were significantly increased in the PRP-containing groups. The vascularization of HUVECs and polarization of macrophage to the anti-inflammatory M2 phenotype were observed. For in situ bioprinting, the bioinks were printed on the dorsal wound of rats. The printed skin tissues supported high-quality wound closure, modulated the inflammation, and initiated angiogenesis. Because patients autologous PRP and cell suspension could be easily obtained during the operation, the clinical potential of this method could be emphasized.

**Figure 6:**
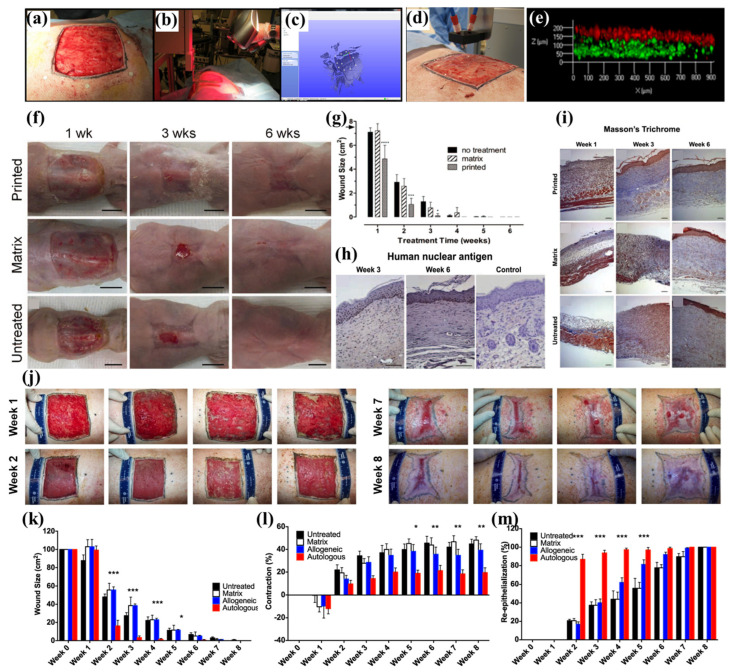
In situ bioprinting of autologous skin cells for healing of full-thickness wounds. (**a**) The full-thickness wound on the porcine skin. (**b**) Geometric information was obtained by 3D scanner. (**c**) Scanned data with its coordinate system. (**d**) In situ printing process. (**e**) The layering of FBs (green) and KCs (red) in the skin constructs. (**f**) Observation of wound healing on the mouse model using printed, dressing matrix, and untreated control for 6 weeks. (**g**) Size of the wound for 6 weeks. (**h**) Anti-human nucleus shows that human cells were maintained for 6 weeks. (**i**) Masson’s trichome staining showed fast re-epithelialization in the printed group compared to the dressing matrix and untreated control. (**j**) Observation of wound healing process in the porcine model. (**k**) Wound size, (**l**) contraction, and (**m**) re-epithelialization, during 8 weeks, show that the printed skins containing autologous cells induced excellent wound healing. The asterisks (*~***) refer to significant differences (* = *p* < 0.05; ** = *p* ≤ 0.01, and *** = *p* ≤ 0.001). The data were reproduced from Ref [105] Copyrights 2018 Nature Publishing Group.

In the other study conducted by Zhao et al., a mobile skin bioprinting system was reported that provides rapid on-site management of extensive wounds (Figure 6) [105]. Fibrin and thrombin-incorporated Col I bioinks were synthesized and FBs and KCs were incorporated. After scanning the geometric information of the wound site, FBs-containing dermal bioink and KCs-containing epidermal bioinks were subsequently printed on the murine and porcine full-thickness excisional wounds. During 6 weeks of observation on mice, printed constructs facilitated the wound healing process and had increased cellularity. The printed tissues on the porcine skin showed a decreased wound size and contraction, with increased re-epithelialization, especially in the autologous cell-laden bioinks. The autologous bioinks showed the formation of rete ridges, keratinized stratified squamous epithelium, and proliferating KCs, as well as the formation of woven dermal Col, and regularly distributed vasculature. Therefore, the combined application of autologous cells and the developed bioinks for the in situ bioprinting system are suggested to support the rapid healing of full-thickness wounds.

Insufficient nutrients and oxygen supply in the transplanted skins often become a critical hurdle in the clinical application of in situ bioprinting-based wound healing approaches. Wang et al. introduced oxygenic photosynthesis unicellular microalga (*Chlorella pyrenoidosa*) into the GelMA bioinks, to promote sustainable oxygen under light illumination [106]. The encapsulated living microalgae in the fabricated scaffolds showed an effective and controllable oxygenation capacity to support proliferation, migration, and differentiation of FBs and HUVECs in hypoxic conditions. The skin grafts were degradable in a physiological microenvironment (in DPBS) suggesting their feasibility in the in vivo application. Further in situ bioprinting on dorsal skins of rats showed facilitated wound closure with increased re-epithelialization, as well as Col synthesis and angiogenesis. As emphasized in this study, adequate covering of the wounds with varying depths or topographies should be considered for efficient wound closure. Rauf et al. developed self-assembling ultrashort peptides to apply for in situ bioprinting, allowing the deposition of cells under physiological conditions [107]. The ultrashort peptides, namely IVZK and IVFK, were fabricated and showed efficient physicochemical durability, common viscoelastic property of hydrogels, and biocompatibility. One highlight is that the peptide bioinks provided sufficient nucleation sites for silver ions to form the siliver nanoparticles, inside the 3D construct. The separated inner flows with two different mixing zones enabled prevention of premature mixing of the peptides between buffer and internal clogging of the nozzle, to achieve high resolved in situ bioprinting in a physiological condition. During 21 days of in vitro culture, laden HDFs showed promoted growth and proliferation both in IVZK and IVFK bioinks, which is comparable to or even higher in Alg-Gel bioinks. The expression of angiopoietin Like 4 (ANGPTL4), thrombomodulin (THBD), and CITED2 were also up-regulated in the IVZK and IVFK bioinks, which are essential factors for the proper functioning of FBs. Although none of the in vivo tests were assessed in this study, the novel 3D printing system and self-forming bioinks, specialized in physiological bioprinting, could be applied for future research in wound regeneration.

## 5. Conclusions and Future Perspective

The development of tissue-engineered skin based on 3D bioprinting is one of the most highlighted strategies for their vigorous application as wound healing grafts. Three representative 3D printing methods that are vigorously applied for bioprinting include EBB, LAB, and DBB methods. Because natural polymers and synthetic polymers have advantages as bioink materials, a novel combination of them could enhance biocompatibility, 3D printability, and biofunctionality. We summarized recent advances and innovations of 3D bioprinted skin following subsections such as bioink materials, developed structures, and functions. Some papers are included in two or more sections but were categorized based on the most emphasized topics. Natural hydrogels such as Alg, CS, Gel, Col, fibronectin, and HA feature high cytocompatibility, and most of them provide a natural niche to support cell behaviors such as spreading, adhesion, migration, and proliferation (especially for ECM-derived materials). The dECM materials retain molecular compounds from the native tissues such as growth factors, proteins, and fibrous materials. They support constructive and site-specific remodeling responses without host inflammatory and immune responses. Synthetic hydrogels can have versatile properties through chemical modifications, and their excellent elasticity or stiffness enable 3D printing of bulk constructs. Meanwhile, the structural development of skin includes hair follicles, vasculature, and full-thickness models. The hair of the skin supports barrier function against UV light and pathogens, as well as maintenance of body temperature and hygiene. To ensure the long-term survival of the skin grafts without necrosis. Vasculature is an essential factor to provide nutrients and oxygen. The full-thickness skin is the most highlighted part that recapitulates multi-layer structures and appendages. Using this model, not only full recovery of the wound site but also skin-on-a-chip for substitution of the animal study is suggested. New skin models with specific functions have been developed. The antimicrobial skin models prevent infections during and after transplantation, while the disease model can be utilized for the study of mechanism and drug screening tools. The in situ bioprinting technology deposits living skin tissue on the defective areas by directly printing bioink, which provides significant freedom in the operation process.

Through the evaluation of printability, efficiency, and therapeutic effects, transplantation of the 3D bioprinted skin is proposed as a promising strategy for skin wound repair with the potential for clinical translation. The final goal of 3D bioprinted skin is to recapitulate biomimetic ECM that serves as structural support for skin cells, and stimulates them to differentiate into mature skin tissues, that closely match native skin with the stratified epidermis, dermis, and hypodermis with blood vessels, nerves, muscles, and skin appendages. However, there are some more issues to be resolved as follows. (1) Verifying the advantages of bioprinting skins over conventional grafts on large animal models and human trials with long-term observation. (2) Developing novel bioink materials with tunable mechanical, viscoelastic, and apathogenic properties, while maintaining biocompatibility and biofunctionality for wider applications. (3) Searching for new solutions for laden cells expressing insufficient phenotypes, for developing skin appendages such as hair shaft, sweat gland, and sebaceous gland to retain the functions of the native skin. Recent studies showed that ESCs and skin-derived precursor cells can be induced from the iPSCs, implying a large number of skin cells can be harvested from a new source [122,123]. (4) Technical development in 3D printing fidelity and reproducibility. Further development of printing technologies could accelerate fabrication times, to ensure clinically relevant scaled functional skins with high cell viability. 

With advances in operational imaging techniques using CT or MRI, 3D printed prostheses could meet the personalized structures that match the patient’s anatomy and injury needs, proposing the clinical efficiency of 3D bioprinting technologies. However, most of the contemporary medical applications of 3D printing is limited to non-living constructs, for structural support or space-filling prostheses. This can be attributed to the regulatory challenges that are proposed for the clinical application of functional 3D bioprinted constructs containing living cells and bioactive materials. The food and Drug Administration (FDA) currently assesses 3D printed medical devices and conventionally made products under the same guidelines [124]. 3D bioprinted skins are intrinsically different from other grafts in terms of the complexity of mechanisms and has yet-unknown long-term effects in human hosts. The several active components and manufacturing parameters may have large and unpredictable effects on the human body reactions, which makes it hard to meet the guidelines and the regulatory processes proposed. In addition, there is an urgent need for guideline standardization for bioprinting, because no standards for 3D bioprinting (i.e., process, bioink materials, and cells) have yet been established. To address the issues, more inventive studies and clinical trials should be conducted to clarify the long-term effectiveness and side effects. Standardized culture conditions and quality control can ensure product safety, efficacy, and reproducibility. We expect that researchers with interdisciplinary backgrounds will advance the field of 3D bioprinting for skin regeneration, by reflecting on the problems and the concerning suggestions.

## Figures and Tables

**Table 1:** Material advances in 3D bioprinting of skin.

Classifications	Materials	Cell Components (Origin)	Printing Methods	Printability (Fidelity) and Scalability	Biological Assessment of Printed Constructs	Advantages (+) Challenges (−)	Ref.
Natural hydrogel	Alg and Gel	AECs (human term placenta) and WJMSCs (human umbilical cord)	Extrusion	- Precision in 151 ± 13.04 μm-grid - Millimeter-sized tube, cylinder, box, nose, and ear are printable.	- Microarray analysis on global gene expression - rtPCR on verification - CCK-8 and live/dead assay on cell viability and proliferation	+ The bi-layered skin-like constructs were fabricated using AECs and WJMSCs. + High printing precision (151 ± 13.04 μm) and structural fidelity were achieved. + The expression of genes relating to re-epithelization and wound healing was significantly increased. − Limited cell adhesion and spreading.	[46]
	CS-genipin-PEG	KCs (human epidermis) and HDFs (human dermis)	Extrusion	Square disk	- MTT assay on the viability - Live/dead assay	+ The low printing pressure (20—40 kPA) induced high cell viability. + The genipin-based crosslinking maintained high cell viability (93%). − Low printing fidelity and cell spreading.	[47]
	PECMA	HNDF (human neonatal foreskin)	Extrusion	Millimeter-sized few-layered lattice.	- Live/dead assay - IF staining on ECM production	+ Dual crosslinking system using UV radiation and calcium-mediated ionic gelation was achieved. + The bioink supported cell growth and de novo deposition of ECM components of the dermal tissue. − Low printing fidelity	[48]
dECM	dECM (goat)	L929 cells (murine connective tissue)	Extrusion	2 × 2 cm construct with micron-sized lattices	- IF staining on cell morphology and alignment	+ H/H NaCl solutions-based decellularization method showed a much high yield and maintained high residual DNA and ECM contents. + The shear thinning property of bioink induced. − Detailed study for trypsin protocol is required.	[49]
	Porcine dECM and fibrinogen	HDFs (human dermis)	Extrusion	Various geometry at millimeter scales	- H & E staining - Live/dead assay	+ The dECM components strengthen the mechanical and shear thinning properties. + The long-term viability of laden FBs was significantly increased by dECM incorporation. − Cell-level biological assessments are required.	[50]
	Microfat clusters (from human) and ColMA	MSCs, ASCs, and EPCs in human microfats	Extrusion	2 cm lattice disk with 1.5 mm line fidelity	- ELISA on wound healing cytokine - AlamarBlue assay on cell metabolic activity	+ The developed fat processing system can process lipoaspirates into microfat clusters comprising highly viable cells preserved in a native niche. + The expression of proinflammatory and anti-inflammatory cytokines suggests the wound healing microenvironment. − Periodic changes of microfats and bandages are required.	[51]
	DSIS slurry (porcine)	FBs (rat normal skin)	Extrusion	80-layered 2 cm-high lattice constructs with micron-sized lattices	- Live/dead assay - Nucleus staining - MTT assay on proliferation - Western blotting and rtPCR on vasculature and ECM production	+ Highly fine lattice structure was built up to 80 layers (~2 cm in height). + Enhanced FBs behaviors and production of ECM proteins including Col I, Col III, and fibronectin. − Hydration is required for cell-laden printing.	[52]
Synthetic hydrogel	PVA, agarose, nanocellulose, and Alg	HFBs and HUVECs (human umbilical cord)	Extrusion	Tissue-scale human face skin	- Live/dead assay - Alamar blue on proliferation - Biochemical analysis of DNA and Col contents - ELISA and IF staining on angiogenic markers	+ The tissue-engineered faces were fabricated with customizable shapes and sizes that can be vascularized. + The collapsing problem of hydrogel bioink in tissue scale constructs could be solved by the addition of PVA sacrificial layers. + Continuous vascular-like structures were developed with vasculatoid phenotypes. − Fast 3D printing is required for large structure fabrication.	[53]
	PVP and Col	HDFs	Droplet printing	Controllable and layered arrays of micron-sized drops	- Live/dead assay - PrestoBlue assay on proliferation and spreading	+ A hierarchical porous Col architecture was recapitulated similar to native skin. + A novel printing method with a controlled number of droplets with layer deposition was accomplished. − Further biological assessments need to be progressed.	[54]
	PCL, SS, CS, and Alg	NHDF	Extrusion	Layered lattice structure	- MTS and live/dead assay on the viability - dsDNA assay on proliferation - CLSM on cell migration - Anti-bacterial property	+ The combination of electrospun nanofibrous matrices and 3D bioprinted constructs mimicked the epidermis-dermis structures. + The nanofiber layer provides antibacterial property while the printed layer confers a moist environment. − Long-term observation is required for full fusion of dermal-epidermal layers.	[55]

Abbreviations: AECs, amniotic epithelial cells; Alg, alginate; ASCs, adipose stromal cells; CCK-8, cell counting kit-8; CLSM, confocal laser scanning microscopy; ColMA, Col methacryloyl; CS, chitosan; DNA, deoxyribonucleic acid; DSIS, decellularized small intestinal submucosa; ELISA, enzyme-linked immunosorbent assay; EPCs, endothelial progenitor cells; Gel, gelatin; HDFs, human dermal fibroblasts; HFBs, human fibroblasts; HNDF, human neonatal dermal fibroblasts; HUVECs, human umbilical vein endothelial cells; H&E, hematoxylin and eosin; IF, immunofluorescence; MSCs, mesenchymal stem cells; NHDFs, normal human dermal fibroblasts; PECMA, pectin methacrylate; PIL, poly(ionic liquid); Pr, printability; PVA, polyvinyl alcohol; PVP, Polyvinylpyrrolidone; QCS, quaternized chitosan; rtPCR, real-time polymerase chain reaction; SS, silk sericin; and WJMSC, Wharton’s jelly derived mesenchymal stem cells.

**Table 2:** Structural advances in 3D bioprinting of skin.

Classifications	Materials	Cell Components (Origin)	Printing Methods	Printability (Fidelity) and Scalability	Biological Assessment of Printed Constructs	Advantages (+) Challenges (−)	Ref.
Hair	GelMA and HAMA	NHDFs (human dermis), HaCaT cells (human epidermis), and HFDPCs (human scalp)	Extrusion	Triple-layered micron-sized lattice structure	- Live/dead assay - IF staining on hair-inducing properties and skin morphology	+ A papillary layer was recapitulated by 3D printing. + Enhanced epidermis-dermis interaction supported spontaneous hair pore development. − Long-term observation is required for full hair shaft development.	[23]
	GelMA and rhCol3	HaCaT cells and HDFs (human skin)	Extrusion	Filament fusion test at millimeter scales	- Live/dead assay - CCK-8 assay on proliferation - rtPCR on cytoskeletons and ECM production - IHC analysis on epithelialization and in vivo wound healing properties	+ rhCol3 enhanced the growth of HaCaT cells and HDFs. + Enhanced wound healing and hair follicle development on in vivo rat model. − higher cell spreading and population in dermal layers are required.	[78]
	Col	KCs (human neonatal foreskin dermis), HUVECs (human umbilical cord), and HFDPCs (human scalp)	Extrusion	Micropillar mold 500 μm in diameter and 4 mm in length	- IF staining and IHC on HFU development and vasculature both in vitro and in vivo	+ The HFU-developed and vascularized dermal constructs were fabricated. + The skin engraftment allows for human hair growth in nude mice. − Reproducibility on hair shaft protrusion should be confirmed.	[79]
Vascularization	PGA, and xeno-free dermal and epidermal bioink	HECs (umbilical cord blood), FBs (human dermis), PCs (human placentas), and KCs (human epidermis)	Extrusion	NA	- Flow cytometry on cell phenotypes - IF staining and IHC analysis on tissue structures	+ A mature stratified epidermis with rete ridge-like structures was developed. + The developed microvessels prevented graft necrosis and induced perfusion with host microvessels. + A xeno-free approach to complex tissue engineering was achieved. − Further studies are required on the efficacy of the xeno-free strategy and the degree of wound bed contraction.	[80]
	GelMA, SCS, and DA	BMSCs, HUVECs, NHDFs, and HaCaT cells (from human origin)	Extrusion	Lattice-structured constructs	- Hoechst staining on the viability - IF staining on angiogenesis - ARS staining on osteogenesis - Cell scratch assay on wound healing	+ Micro-vascularization as tubelike structures with endothelial cell marker expression were confirmed. + Enhanced in vitro skin wound healing activity and maintained multipotency of BMSCs. − Further biological evaluations are required.	[81]
	GelMA, HA-NB, and LAP	HFBs and HUVECs (from human origin)	DLP	Cylinder with submicron lattices	- Live/dead assay - TIANamp Genomic DNA Kit on cell proliferation - IF staining on cell tracking, migration, and adhesion - IF and IHC analysis on inflammatory cell infiltration, wound healing, and angiogenic markers expression.	+ The DLP enabled interconnected microchannel formation that facilitates cellular behaviors. + Efficient neovascularization was achieved by mimicking the physiological structure of native skin. + Induced instant defense function and dermal regeneration with skin appendages in large animals. − In-depth studies on underlying mechanisms in the hair follicle and blood vessel regeneration are required.	[82]
	Rat tail Col I	HFBs, HUVECs, HECFCs, PCs, and HKCs (from human origin)	Extrusion	NA	- IF staining and IHC analysis on skin structure, epithelialization, ECM production, and vascularization.	+ In vitro, HKCs formed a multilayered barrier, while the HUVECs and PCs self-assemble into interconnected microvascular networks. + Transplantable skin grafts composed of an irrigational microvascular system were developed. − Harvesting plenty of healthy cells from the patients are required.	[83]
Full thickness	Gel, glycerol, and HA	KCs, dark melanocytes, HDFs, HFDPCs, HDMECs, and preadipocytes (from human origin)	Extrusion	2.5 × 2.5 cm triple-layered patch with micron-sized lattices	- Picrosirius red staining for Col fiber - IHC analysis on structural maturation	+ Epidermis-dermis-hypodermis triple−layered skin mimetics were 3D bioprinted. + Matured normal and basket weave Col was observed. − Immune responses in the large animal models should be elucidated.	[84]
	GelMA and Alg	HDFs (human dermis), HUVECs (human umbilical cord), HKCs	Extrusion	Micron-sized lattice	- Live/dead assay - IF staining on cell morphology and proliferation - ELISA on ECM synthesis and migration	+ A 3D full-thickness skin model composed of epidermis-dermis with vasculature was fabricated. + Controlled matrix stiffness regulated pro−Col1α1 and MMP-1 expression. + Repeated HKCs seeding and Gel coating support epidermal differentiation. − Epidermal markers should be further elucidated.	[85]
	Alg, Gel, and DCEL	FBs (human dermis) and KCs (human epidermis)	Extrusion	Micron-sized highly fine lattice-structured cylinder	- MTT assay on cytotoxicity - Live/dead assay - IF staining on Col and keratin expression.	+ The incorporated DCEL can induce the uniform distribution of cellulose fibers within bioinks. + The distinct epidermal-dermal histological features were visualized with specific marker expressions. − Further biological assessments should be conducted.	[86]
	Col	HDFs (human neonatal dermis), HKCs (human neonatal epidermis), and MCs (human darkly pigmented neonatal epidermis)	Extrusion	2 × 2 cm stratified constructs	- IHC analysis on skin structures and differentiation	+ KC formed the stratum corneum and freckle-like pigmentations were developed by MCs at the dermal-epidermal junction. + First developed engineered ephelides in biomimetic skin. −In−depth studies for melanin production and pigmentation should be conducted.	[87]
	Gel, Col I, elastin, fibrinogen, laminin, and entactin	HDF (neonatal human dermis) and HKCs (neonatal human epidermis)	Extrusion	Directly 3D printed on a well plate.	- H&E staining on epidermal differentiation - IHC analysis on the tight junction,—ECM proteins, and proliferation markers. - MTT assay on the viability - OCT on tissue morphology	+ Four primary layers of the epidermis were developed. + Stratum granulosum formed f-TKD shape allowing homeostasis by tight junction barrier. − Need to apply iPSCs to obtain consistent and reproducible KCs sources.	[88]

Abbreviations: ARS, Alizarin red S; BMSC, bone marrow-derived stem cells; DA, dextran aldehyde; DCEL, diethylaminoethyl cellulose; DLP, digital light processing; GelMA, Gel methacryloyl; HAMA, hyaluronic acid (HA) methacryloyl; HA-NB, NB-linked HA; HDMECs, human dermal microvascular endothelial cells, HECFCs human endothelial colony-forming cells; HECs, human endothelial cells; HFDPCs, hair follicle dermal papilla cells; HFU, hair follicle unit; HKCs, human KCs; IHC, immunohistochemical; iPSCs, induced pluripotent stem cells; LAP, lithium phenyl-2,4,6-trimethylbenzoylphosphinate; MCs, melanocytes; MMP, matrix metalloproteinase; NB, N-(2-aminoethyl)-4-(4-(hydroxymethyl)-2-methoxy-5-nitrosophenoxy) butanamide; NHEKs, normal human epithelial KCs; OCT, optical coherence tomography; PCs, placental pericytes; rhCol, recombinant human Col; SCS, succinylated chitosan; and SKPs, skin-derived precursors.

**Table 3:** Functional Advances in 3D Bioprinting Skin.

Classifications	Materials	Cell Components (Origin)	Printing Methods	Printability (Fidelity) and Scalability	Biological Assessment of Printed Constructs	Advantages (+) Challenges (−)	Ref.
Antimicrobial	GA, MeC, and Alg	HFBs (human foreskin)	Extrusion	Square (h = 4 mm) and round (h = 13 mm) disk with micron-sizes inner lattices.	- Antibacterial activity - Live/dead assay	+ Feasibility of complex 3D assemblies fabrication due to the high thixotropic behaviors. + Gallium components exhibited antibacterial activity toward *S. aureus* and *P. aeruginosa*. + The bioink facilitated FB behaviors. − Further assessments of skin functions of the constructs are required.	[98]
	HA-MA, HA-SH, and Nafcillin	HDFs (human dermis)	Extrusion	Millimeter-sized lattice (1 × 1 cm)	- Live/dead assay - CCK-8 assay on cell proliferation	+ High swelling ratio and a high controlled degradation rate according to the ratio of HA-SH and HA-MA. + Sustained Nafcillin release enabled anti-bacterial activities. − Future studies on disease models are required.	[99]
Disease	Decellularization of the porcine dermis, hypodermis, and vascular tissues.	NHDFs, dHDFs, NHPAs, dHPAs, HEKs, and HUVECs (from human origin)	Extrusion	Hypodermis-dermis-epidermis triple layers with microfluidic channels	- IF and IHC analysis on epithelialization, ECM production, vasculature, and tissue viability. - rtPCR on diabetic markers - ELISA on inflammatory response - Glucose uptake test	+ Normal and diabetic full-thickness skin model was fabricated. + The normal keratinocytes differentiated as diabetic epidermis through the interaction with the diabetic dermal compartment. + Typical hallmarks of the native diabetic skins were observed. − Efficient co-culture system, densely packed adipose lipid droplets, and sprouting vessels should be further developed.	[100]
In situ bioprinting	Matrigel	ESCs (neonatal mouse skin) and SKPs (neonatal mouse skin)	Extrusion	Adaptive multi-DoF in situ bioprinting at mm-sized lattice	- Cell viability and proliferation test - IF staining and flow cytometry on ECM proteins - ALP staining - rtPCR on stemness and hair genesis - IHC and IF analysis on extracted mouse skin	+ An adaptive bioprinting robot to proceed rapidly in situ bioprinting on a full-thickness excisional wound was developed. + In vivo wound healing including epidermis, dermis, blood vessels, hair follicles, and sebaceous glands was observed. − Development of mechanically stable and apathogenic Matrigel is needed for clinical translations.	[101]
	GelMA and PEO	NIH/3T3 cells (murine embryo) and HUVECs	Hand-held Extrusion	Tailored in situ printing on different scars	- Live/dead assay - IF staining on cell morphology	+ A portable handheld extrusion bioprinter was developed for in situ wound dressing. + The pore-forming bioink enhanced liquid and oxygen transport that supported the growth of laden cells. − Further functionalization is required including multi-bioinks arrangement, replacement of phototoxic UV, and enhanced antimicrobial property.	[102]
	Fibrin and HA	MSCs (human)	Hand-held Extrusion	Directly injected on the wound by soft wheel-based handheld instrument.	- Flow cytometry on MSCs markers. - IHC analysis on MSC differentiation. - Live/dead assay - IF and IHC analysis on tissue morphology	+ The MSCs-containing fibrin sheets are directly delivered to the wound bed, improving re-epithelialization, dermal cell repopulation, and neovascularization. − Strategy for reliable delivery of engineered constructs on large physiological areas should be further investigated.	[103]
	PRP, Alg, and Gel	HDFs, HESCs, HUVECs (from human), and RAW 264.7 macrophages (murine tumor)	Extrusion	In situ bioprinted on rat skin wounds	- Masson staining - IF staining on fibrinogen - CCK-8 assay on proliferation - Scratch assay on migration - rtPCR and IF staining on cell function and ECM production - IF staining and IHC analysis on in vivo printed constructs	+ The PRP facilitated cellular behaviors, tube formation of HUVECs, and macrophage polarization. + For in situ bioprinting, the bioinks modulated the inflammation and initiated the angiogenesis. − Long-term observation in large animal models and elucidation of molecular mechanisms are required.	[104]
	Fibrinogen, thrombin, and Col I	Autologous or allogeneic FBs and KCs	Extrusion	In situ bioprinted on mouse skin wound	- H&E and Masson’s Trichrome staining on tissue morphology. - IHC analysis on vascularization, Col deposition, myofibroblast activation, and cell proliferation.	+ A mobile skin bioprinting system that provides rapid on-site management of extensive wounds was developed. + Autologous FBs and KCs induced rapid wound closure, reduced contraction, and accelerated re-epithelialization. − Long-term tracking of implanted cells is required for the identification of whether regenerated skins consist of bioprinted cells.	[105]
	Alg, GelMA, microalgae	FBs and HUVECs (human)	Extrusion	In situ bioprinted on rat skin wounds	- IF and IHC analysis on wound closure and angiogenesis.	+ Microalgae in the scaffolds showed an effective and controllable oxygenation capacity to support behaviors of FBs and HUVECs in hypoxic conditions. + In situ printed constructs facillitated wound closure, re-epithelialization, Col synthesis, and angiogenesis. − Potential side effects of microalgae in human body should be elucidated.	[106]
	IVZK and IVFK	HDFs	Extrusion	2 × 2 cm lattifce with micron-sized lattices	- Gene expression and IF staining of cell morphologies and funcions	+ Prevention of premature mixing of the peptides between buffer and internal clogging of the nozzle for in situ bioprinting in physiological conditions. + promoted growth and proliferation of HDFs with up-regulation of FB functions-relating genes. − Further studies required for in situ bioprinting.	[107]

Abbreviations: dHDFs, diabetic dermal fibroblasts; dHPAs, diseased human subcutaneous preadipocyte cells; DoF, degree of freedom; DTP, 3,3′-dithiobis(propionylhydrazide); ESCs, epidermal stem cells; Ga, gallium; HA-MA, HA methacrylic anhydride; HA-SH, thiol-modified HA; HESCs, human epidermal stem cells; IVFK, Ac-Ile-Val-Phe-Lys-NH_2_; IVZK, Ac-Ile-Val-Cha-Lys-NH_2_; MeC, methylcellulose; NHPAs, normal human subcutaneous preadipocyte cells; PEO, Polyethylene oxide; PRP, platelet-rich plasma; Ul84, Australian Ulvacean macroalgae.

## Data Availability

Not applicable.
